# Myocardial T1 measurement: comparison of modified Look-Locker inversion recovery (MOLLI) and TI scout in the Multi-ethnic Study of Atherosclerosis (MESA)

**DOI:** 10.1186/1532-429X-14-S1-P265

**Published:** 2012-02-01

**Authors:** Yuan Chang Liu, Chia-Ying Liu, Rob J van der Geest, Songtao Liu, Marcelo Nacif, Joao A Lima, David A Bluemke

**Affiliations:** 1 Johns Hopkins University, Baltimore, MD, USA; 2 Department of Radiology, Leiden University Medical Center, Leiden, Netherlands; 3 Radiology and Imaging Sciences, National Institutes of Health, Bethesda, MD, USA

## Summary

Different cardiac MR acquisition sequences have been used to obtain myocardial T1 values. Among which available techniques, MOdified Look-Locker Inversion-recovery (MOLLI) and inversion-recovery TrueFisp (TI scout) sequences are widely used in research and clinical settings. We compared myocardial T1 values derived from both MOLLI and TI scout techniques in the post gadolinium delayed enhancement experiments in a large-scale multicenter study (Multi-Ethnic Study of Atherosclerosis, MESA). Our results indicated that myocardial T1 measurement using MOLLI is reproducible with TI scout.

## Background

Different cardiac MR sequences have been used to obtain myocardial T1 values. The multipoint approach, as first described by Look and Locker (TI scout), has been widely used for estimation of the optimal TI in the assessment of myocardial delayed enhancement (DE). The other technique, MOdified Look-Locker Inversion-recovery (MOLLI) allows a rapid and highly reproducible T1 mapping of the heart with high levels of intra and inter-observer agreement. Even though each technique claims its own merit and reliability, the post-contrast myocardial T1 value is confounded by various factors that inhibit comparison of myocardial T1 between patients. In this work, we compared myocardial T1 values derived from both MOLLI and TI scout techniques in the post gadolinium (Gd) DE experiments in a large-scale multicenter study (Multi-Ethnic Study of Atherosclerosis, MESA).

## Methods

All cardiac MRI studies were performed using Siemens 1.5T MR scanner. 390 MESA participants without finding of DE were included in the data analysis. Gd (Magnevist; Bayer, 0.15mmol/kg) was administrated as a bolus and single slice T1 determinations at the mid-ventricle short-axis view using MOLLI (11 images within 17 heartbeats) was performed at 12 minutes after injection and followed immediately by TI scout imaging. MOLLI was acquired again at 25 minutes post-Gd. All images were processed off-line using MASS research software with the Levenberg-Marquardt fitting algorithm. Region of interest was manually drawn from the same area in septum. We interpolated 12-minute MOLLI T1 linearly using T1 changes between two MOLLI acquisitions. The T1 value of the 12-minute MOLLI was then corrected based on the imaging time difference. Paired student t-test and Bland-Altman analysis were used to compare the T1 values (p<0.05 for statistical significance).

## Results

**Figure 1:**
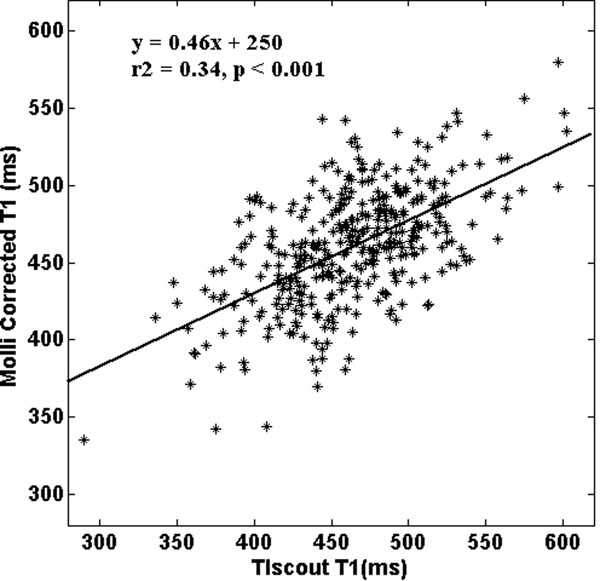
Regression analysis shows good correlation between T1 values acquired by MOLLI and TI scout. The estimated MOLLI T1 values were adjusted for acquisition time difference.

**Figure 2:**
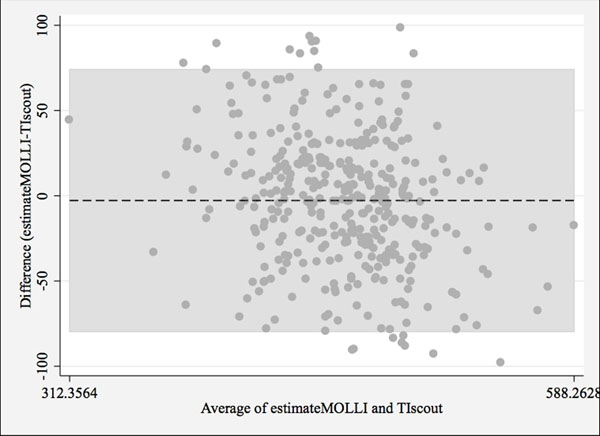
Bland-Altman plot showed good agreement of measuring post-contrast myocardial T1 value from adjusted MOLLI and TI scout. The mean difference is 2.7msec.

The mean T1 values between the post-contrast 12-minute MOLLI and TI scout were significantly different before correction (MOLLI=453.7±37msec, TI scout=463.6±47msec, p<0.001) and was comparable after correction for the acquisition time difference (MOLLI=460.8±37msec, p=0.16). Figure 1 demonstrates the correlation (r2=0.34, p<0.001). Bland-Altman plot (Figure 2) shows good agreement between these two measurements.

## Conclusions

Our results indicated that myocardial T1 measurement using MOLLI is reproducible with TI scout. This evaluation addresses an important issue in which comparison between myocardial T1 derived from different techniques, MOLLI and TI scout, could be possible provided other conditions that affect T1 values such as Gd type/dose and timing remain the same.

## Funding

This study is supported by N01 HC 95168, Multi-Ethnic Study of Atherosclerosis (MESA), National Heart, Lung, and Blood Institute (NHLBI).

